# Do Criminals Live Faster Than Soldiers and Firefighters? A Comparison of Biodemographic and Psychosocial Dimensions of Life History Theory

**DOI:** 10.1007/s12110-020-09374-5

**Published:** 2020-08-22

**Authors:** Monika Kwiek, Przemysław Piotrowski

**Affiliations:** 1 Institute of Applied Psychology, Jagiellonian University, Krakow, Poland; 2 Department of Forensic Psychology and Criminology Institute of Applied Psychology, Jagiellonian University, Krakow, Poland

**Keywords:** Life history theory, Biodemographic indicators, Psychosocial indicators, Life expectancy, Criminal behavior

## Abstract

A high risk of morbidity-mortality caused by a harsh and unpredictable environment is considered to be associated with a fast life history (LH) strategy, commonly linked with criminal behavior. However, offenders are not the only group with a high exposure to extrinsic morbidity-mortality. In the present study, we investigated the LH strategies employed by two groups of Polish men: incarcerated offenders (*N* = 84) as well as soldiers and firefighters (*N* = 117), whose professions involve an elevated risk of injury and premature death. The subjects were asked to complete the Mini-K (used as a psychosocial LH indicator) and a questionnaire which included a number of biodemographic LH variables. Although biodemographic and psychosocial LH indicators should be closely linked with each other, the actual connection between them is unclear. Thus, this study was driven by two aims: comparing LH strategies in two groups of men with a high risk of premature morbidity-mortality and investigating the relationship between the biodemographic and psychosocial LH dimensions. The study showed that incarcerated men employed faster LH strategies than soldiers and firefighters, but only in relation to biodemographic variables (e.g., number of siblings, age of sexual initiation, life expectancy). No intergroup differences emerged regarding psychosocial LH indicators. Moreover, the correlation analysis showed a weak association between biodemographic and psychosocial LH indicators. The results strengthen the legitimacy of incorporating biodemographic LH traits into research models and indicate the need for further research on the accuracy of the Mini-K. The possible explanations for the intergroup differences in LH strategies are discussed.

According to life history (LH) theory, a high level of extrinsic morbidity-mortality that elevates the risk of dying prematurely, in that it is unlikely to be avoided by action undertaken by an individual, favors fast LH strategies (Del Giudice et al. 2015; Ellis et al. 2009). Compared with the general population, both criminal offenders and men working in dangerous professions tend to experience higher rates of morbidity-mortality (Elonheimo et al. 2017; Fisher et al. 2017; Lindberg et al. 2017; Ma et al. 2005), so they might be viewed as more likely to employ fast LH strategies. However, one of the most common motivations among risk-taking professionals is prosocial concern (Firmin et al. 2018; Woodruff et al. 2006), considered to be a component of a slow LH strategy (Rushton 1990; Sherman et al. 2013).

In the current study, we investigate and compare LH strategies in two groups of men at high morbidity-mortality risk but with different levels of prosociality: incarcerated criminal offenders and professionals working as soldiers and firefighters. Since, according to LH theory, the biological and psychological aspects of human functioning are linked to create a consistent LH strategy (Figueredo et al. 2017a), the differences in LH strategies among study groups are measured by investigating both biodemographic and psychosocial LH dimensions. Verifying to what extent biodemographic and psychosocial LH indicators are linked with each other, in order to reflect the premises of LH theory and illustrate a consistent LH strategy in each study group, might be especially important considering the recent debate on the relation between traditionally used biodemographic LH variables (such as number of siblings, age of sexual onset, or life expectancy) and more recent psychosocial LH variables (Copping et al. 2014; Figueredo et al. 2015).

## Life History Theory

LH research theorists are interested in the evolution and nature of the allocation of resources by an individual to various objectives relevant to adaptation, such as growth, body maintenance, and reproduction (Figueredo et al. 2006; Mishra and Lalumière 2008). Given limited energy and material resources, a favorable allocation strategy requires trade-offs, whose nature and degree depend on the current environmental conditions (Del Giudice and Belsky 2011; Ellis et al. 2009; Kruger et al. 2015). The manifestation of these trade-offs is a group of biodemographic variables called LH traits, which include growth rate, age of sexual maturity, duration of pregnancy, child’s birth weight, average number of offspring, duration of breastfeeding, average intervals between consecutive deliveries, the level of parental investment, the rate of aging, and life expectancy (Pianka 1970).

The fact that LH theory traits tend to co-vary across species underlies the key LH theory premise about the existence of consistent LH strategies representing two edges of a LH continuum. Along one edge runs a slow LH strategy, adopted by species characterized by a slow pace of growth, delayed maturation and reproduction, larger body size, low fertility, and high parental investments as well as high life expectancy. In turn, a fast LH strategy typifies species with faster growth, earlier maturation, accelerated reproduction, smaller body size, high fertility, low parental investments, and shorter life expectancy (Promislow and Harvey 1990; see Del Giudice 2019; Del Giudice et al. 2015). The existence of a slow-fast continuum has been found among mammals (Oli 2004; Promislow and Harvey 1990; van de Kerk et al. 2013), birds (Sæther et al. 2004), reptiles (Briggs-Gonzalez et al. 2017), and many other animal taxa (Healy et al. 2019).

Species employing fast LH strategies typically inhabit harsh and unpredictable environments, which are characterized by an elevated level of extrinsic morbidity-mortality in terms of external causes of death and disability that pose a threat unlikely to be avoided or minimalized by action taken by an organism. Under such conditions each individual in a population faces an elevated risk of death, regardless of their age or fitness, which favors accelerated maturation and reproduction (Del Giudice et al. 2015; Ellis et al. 2009). Especially early life adversity tends to accelerate reproductive timing. This phenomenon might be explained by external or internal predictive adaptive response (PAR) hypotheses (Nettle et al. 2013), which are not mutually exclusive. According to the external PAR hypotheses, early adverse experiences may serve as a forecast of future environmental conditions. In this case, the individual should develop an LH strategy that will be adaptive in the anticipated environment. On the other hand, within the meaning of the internal PAR hypotheses, experiencing early-life adversity negatively affects individual survivability at any age. In this context, accelerated reproduction might be caused by the individuals’ prediction of their short life expectancy (Nettle et al. 2013).

Although there are some uncertainties as to whether LH differences between species can be applied to account for individual differences within populations (e.g., Baldini 2015; Zietsch and Sidari 2019), there are arguments supporting such an assumption (see Del Giudice 2019). One strong hypothesis states that individual differences are affected by the same environmental factors (e.g., morbidity-mortality risk) as in the case of cross-sectional variation (Del Giudice 2019). Apparently, aside from environmental influences, individual differences in LH strategies are partly controlled by genes (Bolund et al. 2015; Briley et al. 2017; Tielbeek et al. 2018).

Regarding human LH strategies, they seem to be unique owing to our relatively long lifespan, expended period of children’s dependency on their parents, relatively high male investment in females and offspring, and large brains related to a wide range of psychological attributes (Kaplan et al. 2000) or modern inventions, such as contraception and the welfare state (Figueredo et al. 2015). This unique context makes human LH trade-offs rather complicated and connected with many new psychological traits (Del Giudice 2018). However, before referring to any trait as LH-related, it is important to ensure that it meets several crucial criteria. Namely, LH-related traits should tend to be stable in order to be considered as individual difference variables. They also should co-vary with more objective, traditional LH indicators (i.e., biodemographic LH variables, such as sexual onset) and serve as mediators of LH allocations or at least as proxies of such mediating traits (Del Giudice 2019). Ensuring that these criteria are met could help to avoid confusing traits indicating personality and lifestyle with actual LH indicators, which has been recently pointed out as a genuine issue (see Copping et al. 2014, 2017).

Prototypical sets of fast and slow human LH strategies comprise both biodemographic and psychosocial traits which are likely to co-occur. According to LH theory, a typical fast LH strategy includes biodemographic variables such as early puberty and sexual initiation, becoming a parent at a younger age, having numerous offspring, short intervals between subsequent births, and low life expectancy, whereas a set of psychosocial variables reflecting a fast LH strategy can involve low parental investments, higher levels of aggression, preferences for risk-taking and engaging in antisocial activities (Dishion et al. 2012; Figueredo et al. 2017a). Biodemographic indicators of slow LH strategies include late puberty, postponed sexual initiation, a smaller number of offspring, longer intervals between subsequent births, and high life expectancy, whereas psychosocial traits and behaviors representative of slow LH strategies, involve preferences for monogamous relationships, high parental investments, a tendency for long-term planning, altruism, adherence to the law, and a cautious attitude toward risk-taking (Dishion et al. 2012; Figueredo et al. 2006, 2017a; Rushton 1990).

The division into fast and slow LH strategies is commonly used in the field of LH theory. However, such sets of LH indicators should not be perceived as immutable (Del Giudice 2019). Correlations between LH traits can be complicated and context-dependent; even if some LH traits typically correlate with each other, it does not mean that they constitute a fixed, one-dimensional LH strategy. In fact, many various factors (e.g., the level of one’s physical attractiveness) can affect the correlations between LH traits, possibly leading to the occurrence of many different LH profiles (Del Giudice 2018, 2019). In his extended model of LH-related traits, Del Giudice (2018) introduced some additional profiles to the traditional clusters of LH indicators, pointing out the possibility of multiple fast and slow LH strategies that would comprise different conformations of psychological traits. Such reasoning challenges the currently popular “lumping” approach that posits the existence of fixed sets of LH traits (e.g., Figueredo et al. 2007, 2017b; Giosan 2006).

### Life History Strategies of Men at Risk of Premature Death

As stated previously, the level of morbidity-mortality is one of the key factors influencing the development of a LH strategy (Del Giudice and Belsky 2011; Ellis et al. 2009; Quinlan 2010). When the future is perceived as uncertain, it is more adaptive to pursue immediate gratification than invest in long-term goals, which may not repay the effort. Awareness of the elevated risk of premature death is related to a fast LH strategy and favors, among others, risk-taking behavior (Kruger and Nesse 2006), short-term mating preferences (Dunkel et al. 2010), earlier reproduction and lower parental investments (Quinlan 2010). According to LH theory, extrinsic morbidity-mortality experienced in early childhood has the most significant impact on LH strategy (Belsky et al. 1991; Chisholm et al. 1993; Quinlan 2003). However, it has also been claimed that, due to behavioral flexibility, the pace of LH strategy can be adjusted to environmental life expectancy cues, not exclusively during childhood, but throughout the course of adult life as well (Dunkel et al. 2010; Quinlan 2010).

The tendency to engage in criminal activity is commonly associated with low life expectancy (Aalsma et al. 2016; Lindberg et al. 2017). There are three models of explanation for premature mortality among offenders that are not mutually exclusive (van de Weijer et al. 2016). First is the direct causal model, according to which criminal activity itself increases the hazard of premature death through the possibility of being killed or seriously injured during confrontation with the police, other offenders, or victims. Next is the indirect causal model, which explains earlier mortality in offenders as a result of a criminal lifestyle, including excessive drinking, using drugs, or experiencing adversity, which might lead to health problems and increase the suicide rate. The third explanation is the spurious model, which denies any causal relationship between offending and premature mortality, assuming instead that both earlier mortality and criminal activity are caused by other factors (e.g., personality, self-control, or socioeconomic background; van de Weijer et al. 2016). Although all these models can account for some cases of premature deaths among offenders, many recent studies have indicated a lack of direct connection between early mortality and breaking the law (Tremblay and Paré 2003; van de Weijer et al. 2016; Zane et al. 2018). Most of all, compared with the general population, offenders are more likely to die from unnatural causes (Chen et al. 2010; Elonheimo et al. 2017) such as accidents (Lindberg et al. 2017; Rosen et al. 2008; Zane et al. 2018), suicide (Björk and Lindqvist 2005; Lindqvist et al. 2007), and as a result of substance abuse (Bukten et al. 2017; Forsyth et al. 2014). Moreover, an analysis of data obtained from the National Longitudinal Study of Adolescent Health has shown that adolescents with a pessimistic outlook on their life expectancy are more likely to engage in criminal activity later in life (Nedelec and Beaver 2012). This seems to be in line with LH theory premises, according to which low life expectancy is a cause of, rather than being caused by, delinquency.

With regard to other LH traits, many studies consistently indicate that criminals tend to employ fast LH strategies. In a meta-analysis of approximately five hundred research results, Ellis (1988) showed the link between criminal behavior and a number of fast LH strategy components, such as gender (male), age (12–30), upbringing in a single-parent household, trauma experienced in childhood, early sexual maturation, early age of sexual initiation, promiscuity, low investments in offspring, and anticipation of early death. More recent studies support these findings, indicating a connection between criminal activity and receiving low parental investments in childhood (Hoeve et al. 2009), upbringing in a single-parent household (Barber 2004, 2007; Bartol and Bartol 2014; Minkov and Beaver 2016), high fertility, high sexual drive, promiscuity (Boutwell et al. 2013; Nedelec and Beaver 2012; Yao et al. 2014), early paternity (Lehti et al. 2012; Stouthamer-Loeber and Wei 1998), or making low investments in partners and offspring (Yao et al. 2014). Furthermore, the results of research by Mishra et al. (2017) have shown the association between a fast LH strategy measured by the Mini-K and a higher probability of being arrested, charged, convicted, and incarcerated.

Much less is known about the LH strategies prevalent among men occupied in dangerous professions, and study results related to this issue do not seem to be as consistent as those regarding the LH strategies employed by offenders. Rucas and Miller (2013a) have found that firefighters experiencing low sleep quality and/or quantity are more likely to exhibit fast LH strategy components, such as an elevated propensity for intergroup competition (e.g., by reporting a willingness to support or defend a favorite sports team when at risk of a violent conflict with fans of an opposing team), high investments for mating (e.g., by a tendency to have unprotected sex with a casual partner), disposition to engage in environmental challenges (e.g., readiness to explore unknown places; Rucas and Miller 2013a), increased impulsivity (Miller and Rucas 2012), and external locus of control (Rucas and Miller 2013b). Although sleep disturbance is simply a state and apparently cannot be treated as if it were a LH-related trait, many studies indicate a high prevalence of various sleep problems among both firefighters (e.g., Abbasi et al. 2018; Billings and Focht 2016; Smith et al. 2019) and soldiers (e.g., Collen et al. 2012; Danker-Hopfe et al. 2017; Luxton et al. 2011), which might, in fact, mean that many of these men have increased chances of employing fast LH strategies.

Another important factor which seems to support the assumption that men who work in dangerous professions are likely to employ fast LH strategies is the high morbidity-mortality rate among soldiers and firefighters. Specifically, soldiers are found to be at increased risk of developing depression, posttraumatic stress disorder (PTSD; Grieger et al. 2006; Hoge et al. 2008), and obesity (Bakalar et al. 2018), which can noticeably and negatively affect life expectancy and quality of life in general. In the context of more proximate risk factors, military physical training causes a larger number of injuries (Smith and Cashman 2002), to say nothing of dangerous combat exposure (Grieger et al. 2006; Hoge et al. 2008). Beyond injuries and other physical problems, melee combat experience generates an acute, intense stress response (Clemente-Suarez et al. 2018). Even among psychologically healthy soldiers, the intensity of combat experience tends to lead to increased attention to threat (Ranes et al. 2017). As a result, high exposure to stressful events appears to elevate the risk of suicide among military personnel (Griffith and Bryan 2016) and due to the accessibility of lethal weapons (e.g., firearms), suicidal attempts are more likely to be fatal in the military than in the general population (Anestis and Bryan 2013). Similarly, firefighters are an occupational group with a high exposure to traumatic events (Jahnke et al. 2017) and elevated suicidal risk, mediated by PTSD and depression (Martin et al. 2017). Moreover, firefighting seems to be linked with increased odds of dying from various types of cancer (Daniels et al. 2014; Ma et al. 2005), as well as cardiovascular diseases, caused by many factors, such as shift work (Puttonen et al. 2010), stress and overexertion (Sen et al. 2016), obesity, hypertension, hyperlipidemia, exposure to smoke, and high levels of toxic chemicals (Banes 2014). Lower vigilance and a tendency to skip safety procedures can also lead to fatality, especially among senior firefighters (Kahn et al. 2017).

As illustrated above, in both soldiers and firefighters, the high risk of premature death can be explained by the direct causal model (death during combat or firefighting) or the indirect causal model (e.g., health problems due to occupational conditions; suicide caused by traumatic experiences). In contrast to offenders, whose elevated risk for premature death is often not linked to criminal activity itself, career soldiers and firefighters seem to be at risk of high morbidity-mortality, which is entirely related to their chosen professions. Considering that the pace of the LH strategy depends on extrinsic mortality, which lies beyond the control of the individual and cannot be reduced by changes in their behavior (Quinlan 2010), such a distinction seems interesting. Men in dangerous professions might be less likely to develop faster LH strategies than offenders because they experience less external morbidity-mortality.

Although life expectancy plays an important role in the context of LH strategy, numerous other factors are also related to it. One example may be prosociality, considered to be a component of a slow LH strategy (Rushton 1990; Sherman et al. 2013). In agreement with LH theory assumptions, offenders tend to display low levels of prosocial concern and empathy (Llorca-Mestre et al. 2017). In contrast, risk-taking professionals appear to be driven primarily by prosocial motives in their professions (Firmin et al. 2018; Grant and Wade-Benzoni 2009; Woodruff et al. 2006), which makes their LH strategies seem to be less consistent. The dichotomy of the antisocial motivation of criminal offenders and the prosocial inducement of men in dangerous professions might be in line with one of the fundamental LH assumptions, according to which different LH strategies are developed in response to the level of harshness and unpredictability experienced in childhood. In the field of criminology, adverse childhood experiences are well-established factors underlying criminal activities (Fox et al. 2015; Stensrud et al. 2018). Offenders with childhood exposure to stress (Whitten et al. 2019), physical and emotional abuse (Baglivio et al. 2014), or neglect (McGuigan et al. 2018) can be perceived as having grown up without social support and values of prosociality, trust, and empathy. This, in turn, might be a key factor explaining the difference between criminals engaging in risky antisocial behaviors and men in dangerous professions who tend to risk their lives for prosocial reasons.

In general, career soldiers and firefighters are similar to offenders in that they seem to be at risk of premature morbidity-mortality; however, their LH strategies are rarely investigated. Moreover, the data concerning possible LH strategies predominant among men in dangerous professions are not as consistent as in the case of the LH strategies employed by offenders.

### Relations between Biodemographic and Psychosocial Indicators of LH Strategy

As stated above, LH theory is an approach derived from evolutionary ecology, in which researchers traditionally study objective biodemographic variables, such as the rate of body growth, average number of offspring, average intervals between subsequent births, level of parental investment, or life expectancy (e.g., Pianka 1970). Over time, Rushton (1985) proposed extending these traditional indicators of LH strategy with indicators of a psychological and social nature, such as intelligence, sexuality, extraversion, compliance with the law or activity level.

Currently, a psychosocial approach is often applied in the study of human LH strategies. In the field of psychology, several psychometric tools are designed to assess LH strategy pace. These measures include the High-K strategy scale (HKSS, Giosan 2006), the Arizona Life History Battery (ALHB; Figueredo et al. 2007), the Mini-K (Figueredo et al. 2006), and K-SF-42 (Figueredo et al. 2017b). The two latter instruments constitute alternative short versions of the ALHB. Since one of the biggest limitations of these measures is their self-report character, some researchers have endeavored to find more observable, behaviorally based tools. This has resulted in the California Q-set measure of LH strategy (Dunkel et al. 2015; Sherman et al. 2013) as well as the LH rating form derived from it (Dunkel et al. 2016). Both these measures turned out to correlate with biodemographic indicators of sexual behavior (Dunkel et al. 2015, 2016).

Of all the LH strategy measures listed above, the most commonly used is the Mini-K (Richardson et al. 2017a), 20-item questionnaire which has six domains combined into one coherent construct that reflects the pace of a person’s LH strategy. Despite its popularity, the Mini-K has recently been widely criticized (e.g., Copping et al. 2014, 2017; Gruijters and Fleuren 2018; Richardson et al. 2017a). As Copping et al. (2014), argue the construct seems to combine too many dimensions, and even if they are interrelated, the relationships between them can be explained by differences in personality and investigated using non-evolutionary models. Moreover, although all six domains purport to comprise a consistent LH strategy, some of them can be seen as predictors (mother/father relationship quality) or mediators (e.g., insight, planning and control), rather than indicators of LH strategy (Copping et al. 2017). Thus, despite good reliability, the Mini-K may not be accurate.

Above all, there is a doubt as to whether the psychosocial aspects of human functioning can be treated as indicators of LH strategies without prior verification of their links with more objective traditional measures (Copping et al. 2014, 2017). Biodemographic variables, despite widespread acceptance, are generally not included in research models concerning psychosocial human functioning from the perspective of LH theory, and the results of a recent study (Hurst and Kavanagh 2017) indicate a lack of significant relationships between the results obtained by the Mini-K and biodemographic variables, such as the number of biological and half siblings and the presence of a stepfather. Likewise, research conducted by Copping et al. (2014) showed that the participants’ scores on the HKSS were not related to such classical LH indicators as pubertal onset and the number of sexual partners.

Although researchers agree that, in terms of analyzing LH strategy, both psychological and biodemographic variables are important, and that relationship between them is worth investigating (Black et al. 2017; Copping et al. 2017; Figueredo et al. 2015), the actual existence and nature of such relationship is unclear. Some argue that since traditional biodemographic variables create the basis of LH theory, omitting them in the analysis might run a risk that the examined psychosocial variables are not indicators of LH strategies but reflect something else, such as personality or lifestyle (Copping et al. 2014, 2017). Others believe that nowadays, due to the widespread availability of contraception or welfare state protection, psychosocial measures do not have to be in line with biodemographic LH indicators and may even be more valid than these traditional variables (Figueredo et al. 2014, 2015).

### The Current Study

In the current study, LH strategies were measured using traditional biodemographic indicators as well as psychosocial indicators in two study groups: imprisoned men and men in dangerous professions (firefighters and soldiers). The research was guided by two aims.

The first aim was to determine whether there are differences in the LH strategies employed by these two groups of men. As stated above, although in comparison to the general population, soldiers, firefighters, and inmates are all considered to experience relatively high levels of morbidity-mortality, we hypothesized that inmates would tend to employ faster LH strategies than soldiers and firefighters.

Another aim was to verify the existence and nature of the relationship between the psychosocial dimension of the LH strategy measured by the Mini-K and a group of biodemographic variables. We asked whether these two types of variables are linked to each other in the way predicted on the basis of LH theory and whether they reflect the LH strategies of the respondents equally. Since the results obtained by the Mini-K scale and traditional biodemographic variables should reflect the same LH strategy, we assumed they also should be correlated with each other within each study group. Consequently, intergroup differences in LH strategies should be consistently reflected by both psychosocial and biodemographic variables. As stated before, the Mini-K includes items regarding both the childhood environment and current LH indicators (Copping et al. 2017). Thus, in order to find out how this psychosocial construct corresponds to its biodemographic equivalents, we compared it with a number of traditional biodemographic variables reflecting both the amount of parental investments that the participants received in childhood as well as direct indicators of their LH strategies. Another reason for incorporating childhood environment indicators into the list of biodemographic LH variables used in the current study was that LH theory assumes a strong connection between early childhood conditions (e.g., the amount of parental investment received) and subsequent development of LH strategy. Yet, similarly to the case of direct LH variables, traditional biodemographic indicators of received parental investments seem to be ignored by researchers, who tend to focus instead on psychosocial indicators of a childhood environment such as the socioeconomic status of the family, harsh parenting, and domestic violence (e.g., Foster et al. 2008; Griskevicius et al. 2011; Mell et al. 2018). Although such variables can constitute a valuable source of information about harshness and unpredictability of early environments, biodemographic indicators of LH strategies adopted by participants’ parents might also make some contribution to LH-based research models as traditional indicators of the possible level of parental investment, which seems to be a significant factor when it comes to evaluating the quality of childhood environment.

## Method

### Participants

The research sample consisted of 201 male participants (*M*_Age_ = 33.50 years, *SD*_Age_ = 8.35): 84 inmates and 117 men working as soldiers (*n* = 32) and firefighters (*n* = 85). The inmates lived in a medium-security correctional institution for reoffenders in the city of Tarnow in southern Poland. The participants had been convicted of mostly violent offenses against people and property. During the first stage of the study, one of the researchers, accompanied by a prison psychologist, visited prison cells in order to inform the inmates about the study and ask if they might be willing to participate. The convicts who consented were then individually invited to the prison psychologist’s office, escorted by a prison guard, to complete questionnaires.

A similar procedure was executed in the context of research conducted on career soldiers and firefighters, although because of the more lenient security procedures, no other member of staff assisted in the study process. The researcher approached individual workers on duty, informed them about the study, and asked if they would care to join in. After consenting, the respondents completed their questionnaires. This part of study was conducted in three institutions: a military unit in Krakow and fire brigade units in Tarnow and Nowy Sacz. All cities are located in southern Poland.

Participation in the study was entirely voluntary and anonymous. All participants provided their informed consent. The respondents were not rewarded for their involvement. They were given two brief questionnaires in a paper and pencil version. On average, the respondents took 10 min to complete the survey, after which they were thanked for contributing.

### Measures

The psychosocial dimension of LH strategy was measured using the Mini-K (Figueredo et al. 2006), a 20-item short form of the Arizona Life History Battery. The Mini-K contains six domains (family social contact and support; friends’ social contact and support; altruism; mother/father relationship quality; insight, planning, and control; intentions toward infidelity; religiosity) (see Richardson et al. 2017a), which are expected to reflect a single factor representing a consistent LH strategy. The results are obtained using 7-point Likert scales (ranging from *disagree strongly* to *agree strongly*). The higher the score on the Mini-K scale, the slower the LH strategy, indicated on a slow/fast continuum. The measure demonstrates good internal consistency (alpha around .70; see Olderbak et al. 2014).

In order to assess the biodemographic variables reflecting LH premises, we created a questionnaire that allowed us to examine some crucial aspects of childhood environment conditions (i.e., the age of the subjects’ parents at the birth of their first child, the number of biological siblings and stepsiblings, intervals between the mother’s subsequent births), as well as the direct participants’ LH indicators (i.e., age at sexual onset, having children, age of becoming a father, number of children, number of women with whom the subjects have children, and life expectancy). The questionnaire includes direct questions (e.g., How old was your mother when she gave birth to her first child? How many siblings do you have? Do you have children? How long do you expect to live?). We decided not to include the onset of puberty because of the high possibility for this variable to be unreliable in a study with male participants. Compared with the easy assessment of age at menarche, the age of pubertal onset in men is much more difficult to ascertain, especially retrospectively (Webster et al. 2014).

### Statistical Analysis

The data were prepared and presented as follows: first the normality test for distribution was used, and because of the relatively large sample size, the Kolmogorov-Smirnov test was chosen. Since there were clear deviations from the normal distribution for all variables, nonparametric tests were used to verify hypotheses. To determine whether there are differences between research subgroups in the measured set of characteristics, series of intergroup comparisons were conducted using Mann-Whitney’s *U* test. The test was chosen based on the significant deviations in the results from normal distribution (in subgroups) and statistically significant (chi-squared test) differences in subgroup numbers (Field 2009). Effect sizes were measured with the Glass rank biserial correlation coefficient. In order to verify the research hypotheses about the relationship between the variables, a correlation analysis was performed. The nonparametric Spearman rho correlation test based on ranks was applied to allow for a good estimation of correlation coefficients in the event of distribution deviation (Field 2009). All analyses were performed using the SPSS statistical package, version 23.0.

## Results

### Descriptive Statistics

In the first step of data analysis, descriptive statistics were calculated (Table 1). Since the measured indicators were expressed on a quantitative scale, the entire spectrum of descriptive statistics is presented: minimum and maximum results obtained, measures of central tendency, measure of dispersion, position measurement, and normality tests of distribution. A measure of the reliability of the main dependent variable (Mini-K result) was measured with Cronbach’s alpha. For a general sample it was completely satisfactory α = .78. However, it was slightly lower for the inmates (α = .74) than for the second group (α = .81).

**Table 1:** Descriptive statistics

	Min–Max	*M*	*SD*	Sk	Kurt	K-S *D*
Total sample (*N* = 201)						
Age	19–75	33.50	8.35	1.18	2.58	.12**
Mother’s first childbirth	16–38	22.81	3.36	.97	1.70	.16**
Father’s first child	18–36	25.38	3.72	.49	.01	.11**
Number of siblings	0–10	2.20	1.68	1.16	2.23	.19**
Intervals between mother’s next childbirths	1–25	4.24	3.06	3.36	17.21	.17**
Number of stepsiblings	0–7	.22	.80	4.90	29.96	.51**
Age of sexual initiation	11–30	17.50	2.87	1.24	2.41	.17**
The age of becoming a father^a^	16–45	24.92	4.59	1.12	3.18	.10**
Number of offspring	0–5	1.18	1.29	.95	.23	.24**
Number of women with whom the subject has children	1–5	1.18	.52	4.36	25.84	.49**
Expected age of death	35–100	72.50	14.99	.12	−.55	.12**
Mini-K	−35–57	24.12	14.17	−.53	.95	.05
Inmates (*n* = 84)						
Age	22–75	36.14	9.97	1.04	1.53	.93**
Mother’s first childbirth	16–38	22.11	3.57	1.71	4.68	.87**
Father’s first child	18–36	25.14	4.38	.60	−.15	.95**
Number of siblings	0–7	2.61	1.61	.60	−.16	.93**
Intervals between mother’s next childbirths	1–25	4.46	3.10	3.94	24.32	.69**
Number of stepsiblings	0–7	.46	1.15	3.29	13.28	.47**
Age of sexual initiation	11–30	16.35	2.50	2.26	10.12	.83**
The age of becoming a father	16–45	23.85	5.04	1.72	4.87	.87**
Number of offspring	0–5	1.36	1.44	.92	−.09	.84**
Number of women with whom the subject has children	1–5	1.34	.71	3.16	13.36	.52**
Expected age of death	35–100	68.93	15.14	.26	−.41	.97*
Mini-K	−35–57	23.05	14.52	−.61	2.48	.96*
Firefighters and soldiers (*n* = 117)						
Age	19–50	31.60	6.34	.41	−.25	.11**
Mother’s first childbirth	17–31	23.31	3.12	.44	−.23	.15**
Father’s first child	18–33	25.55	3.17	.39	−.27	.13**
Number of siblings	0–10	1.91	1.68	1.68	4.93	.21**
Intervals between mother’s next childbirths	1–21	4.06	3.03	2.93	11.84	.70**
Number of stepsiblings	0–3	.05	.32	7.72	66.65	.53**
Age of sexual initiation	12–27	18.33	2.84	.95	.90	.18**
The age of becoming a father	18–41	25.83	3.99	.73	2.84	.93**
Number of offspring	0–5	1.05	1.16	.85	.08	.27**
Number of women with whom the subject has children	1–2	1.05	.21	4.39	17.84	.22**
Expected age of death	40–100	75.06	14.42	.08	−.59	.13**
Mini-K	−17–52	24.89	13.93	−.46	−.27	.08

^a^Analyses for this variable were only for men who were fathers: among inmates there were 53; in the remaining group, 63.

* *p* < .05, ** *p* < .01

### Intergroup Differences: Inmates vs. Firefighters and Soldiers

There was a statistically significant difference between groups in the declared first birth of the participant’s mother, *U =* 3542.00, *p* = .001, *r*_G_ = .27. Firefighters and soldiers declared that their mothers had given birth for the first time (*Mdn* [median] *=* 23, *Mrank* [mean rank] *=* 111.73) significantly later than the inmates’ mothers (*Mdn =* 21, *Mrank =* 84.67). It was also observed that the firefighters and soldiers (*Mdn =* 2, *Mrank =* 89.66) declared a smaller (*U =* 3587.50, *p* = .001, *r*_G_ = .27) number of siblings than the inmates (*Mdn =* 2, *Mrank =* 116.79) and significantly longer time intervals between their mothers’ subsequent births (*Mdn =* 3, *Mrank =* 80.89) than the inmates (*Mdn =* 4, *Mrank =* 95.45), *U =* 3124.50, *p* = .057, *r*_G_ = .17. A statistically significant difference was also noted between the number of stepsiblings, *U =* 4075.50, *p* < .001, *r*_G_ = .17, with the firefighters and soldiers (*Mdn =* 0, *Mrank =* 93.83) declaring a smaller number of stepsiblings than the inmates (*Mdn =* 0, *Mrank =* 110.98) (Table 2).

**Table 2:** Intergroup differences: Inmates vs. firefighters and soldiers

	Inmates (*N* = 84)		Firefighters and soldiers (*N* = 117)				
Dimension	*Mdn*	*Mrank*	*Mdn*	*Mrank*	*U*	*p*	*r*_G_
Mother’s first childbirth	21	84.67	23	111.73	3542.00	.001	.27
Father’s first child	25	95.16	25	105.19	4423.50	.226	.10
Number of siblings	2	116.79	2	89.66	3587.50	.001	.27
Intervals between mother’s next childbirths	4	95.45	3	80.89	3124.50	.057	.17
Number of stepsiblings	0	110.98	0	93.83	4075.50	<.001	.17
Age of sexual initiation	16	73.05	18	120.38	2566.00	<.001	.47
Age of becoming a father	23	47.32	26	67.90	1077.00	.001	.35
Number of offspring	1	106.95	1	96.73	4414.50	.197	.10
Number of women with whom the subject has children	1	66.01	1	53.20	1324.50	.001	.22
Expected age of death	65	87.17	73	110.93	3752.00	.004	.24
Mini-K	23	95.52	27	104.93	4454.00	.258	.09

There was a statistically significant difference between the groups in age of sexual initiation, *U =* 2566.00, *p* < .001, *r*_G_ = .47. The firefighters and soldiers (*Mdn =* 18, *Mrank =* 120.38) had later sexual initiation than the inmates (*Mdn =* 16, *Mrank =* 73.05). In addition, the firefighters and soldiers (*Mdn =* 26, *Mrank =* 67.90) became fathers later than the inmates (*Mdn =* 23, *Mrank =* 47.32, *U =* 1077.00, *p* = .001, *r*_G_ = .35) and were characterized by a smaller number of women with whom they had children (*Mdn =* 1, *Mrank =* 53.20) than the inmates (*Mdn =* 1, *Mrank =* 66.01, *U =* 1324.50, *p* = .001, *r*_G_ = .22).

The firefighters and soldiers (*Mdn =* 73, *Mrank =* 110.93) declared a higher expected age of death than the inmates (*Mdn =* 65, *Mrank =* 87.17, *U =* 3752.00, *p* = .004, *r*_G_ = .24).

There was no significant intergroup difference in the scores obtained using the Mini-K (Table 3).

**Table 3:** The Mini-K and biodemographic variables: total respondents and research subgroups; Spearman rho rank correlation coefficients (*N* = 201)

	Mini-K		
	Total	Inmates	Firefighters and Soldiers
Mother’s first childbirth	.14*	.10	.12
Father’s first child	.15*	.17	.13
Number of siblings	.03	−.07	.12
Intervals between mother’s next childbirths	.05	.12	.05
Number of stepsiblings	−.10	−.16	−.02
Age of sexual initiation	−.01	−.04	−.07
The age of becoming a father	.22*	.32*	.06
Number of offspring	.19**	.30**	.14
Number of women with whom the subject has children	−.05	.03	−.13
Expected age of death	.09	.15	.03

* *p* < .05, ** *p* < .01

### Psychosocial and Biodemographic Variables

Both among the inmates as well as among the soldiers and firefighters, higher scores on the Mini-K were correlated with a later age of becoming a parent (*r*_S_ = .22, *p* = .016), and with a larger number of offspring (*r*_S_ = .19, *p* = .007). Moreover, the older the father of the respondent was when his first child was born (*r*_S_ = .15, *p* = .030), or the older his mother when she gave birth to her first child (*r*_S_ = .14, *p* = .040), the higher the results obtained on the Mini-K scale.

Among the inmates, the respondents who scored higher on the Mini-K became parents at a later age (*r*_S_ = .32, *p* = .019), and also had a larger number of offspring (*r*_S_ = .30, *p* = .005), although the correlation was weaker. There were no such associations in the firefighters and soldiers group.

## Discussion

### Intergroup Differences in LH Strategies

One of the aims of the current study was to examine differences in LH strategies adopted by incarcerated offenders and men working as firefighters and soldiers. As predicted, the inmates turned out to develop faster LH strategies than men in dangerous professions. However, this dependency occurred only in relation to biodemographic variables. The inmates’ mothers gave birth to their firstborn at a younger age and had shorter intervals between subsequent pregnancies than the soldiers and firefighters’ mothers. Moreover, compared with firefighters and soldiers, inmates had more biological- and stepsiblings, experienced their sexual onset earlier, had offspring with more women, and reported lower life expectancy.

All the differences reported above consistently indicate that incarcerated men employ faster LH strategies both in relation to biodemographic factors of mating/parenting trade-offs and regarding life expectancy. Although we expected such differences in the context of LH strategy pace, it is interesting that soldiers and firefighters, whose exposure to physical threats is often a part of their daily work routine, expected to live longer than inmates who are incarcerated, constantly monitored, and therefore protected from many external morbidity-mortality cues. Such findings suggest that the perceived risk of premature mortality might not be a simple reflection of prevailing conditions but may tell us something more about the person. To shed more light on this matter, it is worth referring to the previously described predictive adaptive response (PAR) hypotheses, according to which an individual makes predictions about future environmental conditions (external PAR) or their own life expectancy (internal PAR) based on the level of adversity experienced early in life (Nettle et al. 2013). Thereby, when it comes to an individual’s expected survival, early-life adversity might be even more important than objective current morbidity-mortality risk.

Admittedly, we did not ask the participants about their backgrounds. However, our results showed that compared with soldiers and firefighters, inmates had more biological siblings and stepsiblings and their parents started their families at an earlier age. Considering that larger family size and becoming a parent at a younger age are commonly associated with a family’s lower socioeconomic status (Mace 2014), lower parental investments (Stulp and Barrett 2016), as well as a higher occurrence of harsh parenting (Lee and Guterman 2010) and child maltreatment (Scannapieco and Connell-Carrick 2016), we assume that the inmates from our study may have grown up in more adverse households than men working as soldiers and firefighters. That could make them more perceptive of mortality cues in their current environment. Since the correlation analyses used in this study prevent us from using causal language, the possibility presented above is a mere speculation that requires further investigation.

The possibility of higher early-life adversity among the inmates could also serve as an explanation for commonly known differences in prosociality between our two study groups. In fact, as a recent study has shown, growing up in a disadvantaged environment tends to be negatively associated with further prosociality by leading to lower levels of Honesty-Humility and dispositional trust in others (Wu et al. 2020).

It also seems to be plausible that intergroup differences in prosociality are not only the result of a more adverse childhood and explain the different LH strategies adopted by the subjects, but might also affect these LH strategies. As soldiers and firefighters engage in risky behaviors in the service of society, their professions are respected in their communities and are a source of recognition, status, and prestige (King and Karabell 2003; Martens 2005). Therefore, their everyday life must be far less hostile than prison reality, which is typically characterized by a high prevalence of male aggressive competition and dominance (Kupers 2005) and inflicts considerable harm on inmates (Irwin and Owen 2005). Living under such hostile conditions might consolidate inmates’ fast LH strategies and partly explain their relatively pessimistic outlook on the future.

Furthermore, starting a family at younger age and having more offspring turned out to be characteristic of the inmates as well as the inmates’ parents. These similarities in biodemographic indicators of LH strategies between participants and their parents might have occurred partly due to genetic influence. In fact, LH traits are considered to be to some extent heritable (Briley et al. 2017; Figueredo et al. 2004; Tielbeek et al. 2018), which makes genetic confounding another hypothetical explanation of our results.

Another issue is the lack of intergroup differences in terms of number of offspring. In addition, there was no association between the number of children reported by the subjects and the other biodemographic variables. Among the inmates, however, the more children they had, the slower the psychometric LH strategy they employed. Similar results were reported by Richardson et al. (2017b), who argued that one of the reasons for the existence of such associations that are inconsistent with LH theory might be the prevalence of contraception use, which makes it easier to prevent unwanted pregnancies among more promiscuous individuals who are not interested in starting a family. As a result, slower LH strategists who intend to be parents can have more offspring than people with faster LH strategies, who usually prefer not to have children (Richardson et al. 2017b). Our findings seem to confirm the above line of reasoning because, although the number of children was not a reliable indicator of the subject’s LH strategy, the intergroup differences in the number of both biological siblings and stepsiblings were consistent with LH theory predictions. Considering the upward trend in using birth control methods over the several past decades (Blanc et al. 2009; Sonenstein et al. 1998), it becomes apparent that around thirty years ago, when the subjects were born, contraception use was not as ubiquitous as it is at present, and greater mating effort was certainly more strongly associated with a higher number of children. In fact, as the recent study has shown, modern industrialized populations adopting a slower LH strategy tend to enjoy increased fertility (Woodley of Menie et al. 2017), which as stated above is most likely a result of the fact that slower LH strategists are more interested in parenting in general (Clutterbuck et al. 2014). Basically, all these findings lead us to the supposition that number of offspring was a more accurate reflection of LH pace in the past than it is currently. In our modern societies, employing a slow LH strategy seems to be related to higher parental investment, which does not always require having fewer offspring. Perhaps when optimized to its fullest potential, a slow LH strategy may actually favor both the quality and the quantity of children. After all, although parental investments influence children’s fitness, the more an individual has invested in their offspring, the more they can lose by employing a fast LH strategy. Thus, at least to some extent, a slow LH strategy might become more and more beneficial with each subsequent child. The association between a slower LH strategy and having more offspring may also be explained by the fact that people with slow LH strategies are typically perceived as higher-quality mates (Dillon et al. 2013), so they are more likely to attract partners interested in long-lasting relationship and starting families.

### The Connection between Biodemographic and Psychosocial Indicators of LH Strategy

Compelled by the dispute over the existence and importance of the relations between biodemographic and psychosocial aspects of LH dimension as well as the shortage of research done on this subject, we aimed to examine the associations between the group of traditional biodemographic LH indicators and the psychosocial assessment of LH strategy obtained by using the Mini-K.

In general, higher scores on the Mini-K indicating a slower LH strategy were associated with having parents who had their first child at a greater age, being older when becoming a parent oneself and, counterintuitively, having more offspring (discussed in the previous section). This already weak association between biodemographic and psychosocial LH variables turned out to be even less significant within the groups. Among the inmates, the slower LH strategy measured by the Mini-K was connected with becoming a father at a later age and having slightly more offspring. Among soldiers and firefighters there was no connection between biodemographic and psychosocial LH variables at all. These results seem to suggest a weak and incoherent connection between biodemographic LH variables and psychosocial aspects of LH dimension assessed using the Mini-K. On the other hand, more correlations occurred in the general sample than among the subgroups, which might suggest that some connections may be more likely to occur if the sample sizes were larger.

### Limitations and Future Directions

The current study has several limitations. The first is the exclusive use of correlation analyses, which prevents us from making conclusions about any causal dependencies. Admittedly, the use of correlation analyses was justified by the aims of the study, which included investigating intergroup differences and verifying connections between the two groups of LH indicators. In future studies, analyses concerning causal relationships would have to include indicators of the perceived harshness and unpredictability of early and current environments. Incorporating such variables into research projects would provide us with a more holistic perspective on the LH strategies currently being employed. Thus, future studies on the psychosocial LH dimension should include traditional biodemographic variables, on which LH theory is based, as well as indicators of perceived harshness and unpredictability experienced at different stages of life. This could be achieved by carrying out longitudinal studies with repetitive usage of questionnaires regarding personal convictions about other people and their immediate surroundings (e.g., The World Assumptions Scale; Janoff-Bulman 1989).

The use of a nonrandom selection process might be perceived as another limitation in the context of intergroup differences in the Mini-K scores. The study groups were similar in terms of experiencing higher morbidity-mortality rates on average than the general population. Intergroup differences occurred but only in relation to biodemographic LH indicators. However, comparing the study groups to individuals with a lower morbidity-mortality risk (a control group) might provide us with intergroup differences in the Mini-K as well. In fact, when comparing our subjests’ scores on the Mini-K with the results of the study investigated on a sample of university students (Figueredo et al. 2014), we found that the Mini-K average obtained in our research (*M* = 1.21, *SD* = .71) was significantly lower than the average result for the students (1.41; *t*_200_ = −4.07, *p* < .001). Same results were obtained separately for inmates (*M* = 1.15, *SD* = .73; *t*_83_ = −3.25, *p* = .002) and for soldiers and firefighters group (*M* = 1.25, *SD* = .70; *t*_116_ = −2.56, *p* = .012).

Another point, mentioned previously, is that the lack of connection between biodemographic indicators and the Mini-K in the firefighters and soldiers as well as the very weak connections between these variables in the inmates might be partly affected by the relatively small sample sizes.

Also, because inmates are characterized by a high prevalence of ADHD and comorbid conditions (Ginsberg et al. 2010; Rasmussen et al. 2001), we did not want to include too many questionnaires in the study since this could have lowered the inmates’ motivation to participate. As a result, we chose to restrict the psychosocial LH assessment to the Mini-K, which, as mentioned above, is the most popular psychosocial LH measure considered to be an alternative for the ALHB and K-SF-42 and correlated with the HKSS (Dunkel and Decker 2010). Nevertheless, more comprehensive analyses including all psychometric LH measures in relation to traditional LH variables would offer a valuable verification of the results obtained in the current study.

Finally, our study was limited to the LH strategies of male participants only because of our greater access to men in both study groups. Certainly, future studies including female subjects would be welcome.

## Conclusions

In the current study, the inmates tended to employ faster LH strategies than career soldiers and firefighters. The differences in the pace of LH strategies between these two groups were found in relation to traditional biodemographic LH indicators such as mother’s age at the birth of her first child, intervals between consecutive pregnancies, age at sexual debut, number of women with whom the respondents have children, and life expectancy. These results seem to confirm the proclivity of taking biodemographic variables into account when it comes to creating LH theory research models. Although our current environments differ significantly from the habitats in which human LH strategies evolved, traditional biodemographic indicators still appear to reflect these strategies in an accurate and consistent way. It also seems relevant that the inmates anticipated a lower life expectancy than men for whom the risk of injury, health problems, and death is often part and parcel of their daily work routine. This might suggest that when it comes to LH strategies, subjective perception of the risk of premature death might be more important than the current objective environmental risk.

No intergroup differences occurred concerning the psychosocial LH dimension measured by the Mini-K, and there were few connections between biodemographic and psychosocial LH indicators within each group. Such findings seem to strengthen the argument made by Copping et al. (2014), who noted a necessity to conduct studies in order to find out to what extent biodemographic and psychosocial LH indicators reflect the same LH strategy. The current study showed that biodemographic LH indicators reflected the participants’ LH strategies significantly better than the Mini-K. However, further investigations are needed to verify our results.
